# Short term effect of tetrahydrocurcumin on adipose angiogenesis in very high-fat diet-induced obesity mouse model

**DOI:** 10.3389/fnut.2023.1221935

**Published:** 2023-10-09

**Authors:** Bhornprom Yoysungnoen, Umarat Srisawat, Pritsana Piyabhan, Naphatsanan Duansak, Nattapon Sookprasert, Nakorn Mathuradavong, Natwadee Poomipark, Narongsuk Munkong, Pholawat Tingpej, Chatchawan Changtam

**Affiliations:** ^1^Division of Physiology, Department of Preclinical Science, Faculty of Medicine, Thammasat University, Pathum Thani, Thailand; ^2^Division of Biochemistry, Department of Preclinical Science, Faculty of Medicine, Thammasat University, Pathum Thani, Thailand; ^3^Department of Pathology, School of Medicine, University of Phayao, Phayao, Thailand; ^4^Division of Microbiology and Immunology, Department of Preclinical Science, Faculty of Medicine, Thammasat University, Pathum Thani, Thailand; ^5^Division of Physical Science, Faculty of Science and Technology, Huachiew Chalermprakiet University, Samutprakarn, Thailand

**Keywords:** adipose angiogenesis, obesity, vascular endothelial growth factor (VEGF), matrix metalloproteinase-2 (MMP-2), matrix metalloproteinase-9 (MMP-9), tumor necrosis factor-alpha (TNF-α)

## Abstract

Tetrahydrocurcumin (THC) has been shown to possess anti-angiogenic activities. This study aims to investigate the effects of THC on adipose angiogenesis and expression of angiogenic factors that occurs in 60% high-fat diet-induced obese mice. Male ICR mice were randomly divided into 3 groups: mice fed with a low-fat diet (LFD group); mice fed with very high fat diet (VHFD group), and mice fed with VHFD supplemented with THC (300 mg/kg/day orally) (VHFD+THC treated group) for 6 weeks. Body weight (BW), food intake, fasting blood sugar (FBS), lipid profiles and visceral fats weight (VF) were measured. The microvascular density (MVD), TNF-α, VEGF, MMP-2, and MMP-9 expressions were evaluated. The VHFD group had significantly increased total cholesterol, triglyceride, food intake, BW, VF, VF/BW ratio, adipocyte size and the number of crown-liked structures as compared to LFD group. THC supplementation markedly reduced these parameters and adipocyte hypertrophy and inflammation in white adipose tissues. MVD, TNF-α, VEGF, MMP-2, and MMP-9 were over-expressed in the VHFD group. However, THC supplementation decreased MVD and reduced expression of TNF-α, VEGF, MMP-2, and MMP-9. In conclusion, THC suppressed angiogenesis in adipose tissue by the downregulation of TNF-α, VEGF, MMP-2, and MMP-9. With its effects on lipid metabolism as well as on food consumption, THC could contribute to lower visceral fat and body weight. Overall, our study demonstrated the potential benefit of THC in mitigating obesity and associated metabolic disorders along with elucidated the suppression of adipose angiogenesis as one of its underlying mechanisms.

## 1. Introduction

Obesity is a current global major health problem. Excess body weight increases the risk of several diseases, including hypertension, cardiovascular disease, cerebrovascular disease, type 2 diabetes, and cancer (1–3). Obesity is characterized by adipocyte hyperplasia and hypertrophy leading to an increase of adipose tissue mass (4). Like the growth of cancerous tumors, the growth of adipose tissue requires neo-vascularization process to supply growing adipose tissue with nutrients and oxygen. Moreover, recent data show that adipogenesis and angiogenesis are closely related during adipose tissue development (5). Therefore, the inhibition of angiogenesis in adipose tissue can potentially be a strategy to prevent adipose tissue growth and subsequent obesity.

Vascular endothelial growth factor (VEGF) and its receptor (VEGFR) are attractive targets for anti-angiogenic therapy to reduce obesity as they play an important role in adipose angiogenesis (6, 7). In expanding adipose tissue at the early stages of a high-fat diet (HFD)-induced obesity, expression of VEGF in white adipose tissue (WAT) enhances angiogenesis. A previous study demonstrated that anti-VEGF antibody inhibited not only angiogenesis, but also the formation of adipose/angiogenic cell clusters (8) indicating that VEGF is a key mediator of angiogenesis as well as adipogenesis. The recruitment of inflammatory cells also significantly contributes to adipose angiogenesis as activated macrophages can produce potent proangiogenic factors, such as tumor necrosis factor-alpha (TNF-α), VEGF, fibroblast growth factor-2 (FGF-2), and interleukins including IL-1β, IL-6, and IL-8 (9).

Extracellular matrix (ECM) proteolysis is required for cell migration during blood vessel development and for adipose tissue expansion. Matrix metalloproteinases (MMPs), including MMP-2 and MMP-9, are key factors involved in ECM degradation, and their main actions in adipose tissues include adipogenesis, angiogenesis, and adipose tissue expansion. A previous study showed that MMP inhibitors significantly reduced gonadal and subcutaneous adipose tissue masses in HFD-fed wild-type mice (10). MMP inhibitors also reduced body weight gain in obese mice (11). Moreover, several types of angiogenesis inhibitors, such as TNP-470, and VEGFR-2 inhibitors, have been shown to inhibit adipose tissue expansion in mice (12–14), suggesting that the inhibition of these substances could reduce adipose tissue development. Overall, these findings have provided more information about the possible therapeutic interventions of obesity and obesity-associated disorders by targeting the vascular compartment.

The use of anti-angiogenic herbal medicines for regulating adipose tissue growth has gained interest due to their safety and efficacy in treating obesity. Numerous phytochemicals, such as (−)-Epigallocatechin-3-gallate (EGCG) in green tea (15, 16), Korean red ginseng extract (GE) (17), ginsenosides present in ginseng (18), and Ob-X, a herbal composition comprising (*Melissa officinalis*, *Morus alba*, and *Artemisia capillaris*) (19), have demonstrated potential in regulating angiogenesis and inhibit obesity. Specifically, EGCG has been found to inhibit endothelial cell tube formation by disruption the formation of VEGF and VEGFR-2 complexes (16) and reduce epididymal white adipose tissue weight in mice by inhibiting the expression of genes involved in the synthesis of *de novo* fatty acids (15). GE has been shown to reduce adipose angiogenesis in HFD-induced obese mice and *db/db* mice by downregulating the expression of VEGF-A, MMPs and fibroblast growth factor-2 (FGF-2) mRNA (17, 20). Similar to GE, Ob-X has been found to reduce adipose angiogenesis in HFD-induced obese mice by downregulating the mRNA expression of angiogenic factors such as VEGF-A and FGF-2, as well as matrix metalloproteinases (MMP-2 and MMP-9) (21).

Among various phytochemicals under investigation, polyphenols have gained particular interest from researchers due to their natural origin and biological properties. One such plant that has drawn attention is *Curcuma longa* L., commonly known as turmeric. The root of this plant is widely cultivated in tropical regions of Asia and has a long history of daily consumption without any reported toxicity (22). Turmeric is used as a spice and colorant in numerous food preparations, i.e., curry and in cosmetics and pharmaceutical products. A key bioactive compound present in turmeric is Curcumin (CUR), a major polyphenol that exhibits various physiological and biological activities, contributing to its potential in prevention and management of several diseases. CUR has been shown to exhibit anti-obesity property in both *in vitro* and *in vivo* models (23–25). It suppresses preadipocyte cell differentiation and modulates the expression of key transcription factors involved in adipogenesis and lipogenesis (namely, PPAR-γ and CCAAT/enhancer binding protein α). Furthermore, the supplementation of CUR has been shown to exhibit an anti-obesity effect through the suppression of angiogenesis in mice fed a high-fat diet, mediated by the downregulation of VEGF and VEGFR-2 expression (23). These findings indicate that CUR has potential in mitigating obesity. Nevertheless, despite its potential therapeutic benefits, CUR’s bioavailability is hindered by poor absorption from the gastrointestinal tract and extensive biotransformation by intestinal bacteria (26–28). Consequently, researchers have directed their efforts toward identifying and isolating CUR metabolites or degradation product (29, 30).

Tetrahydrocurcumin (THC) stands out as the main metabolite of CUR. THC is distinct from CUR in that it lacks α, β-unsaturated carbonyl moiety and is off-white in color and stable in phosphate buffer as well as in saline at various pH values (31). Moreover, THC gets easily absorbed through the gastrointestinal tract, suggesting its crucial roles in the biological effects induced by CUR. THC possesses various properties, including anti-oxidant (32, 33), anti-inflammatory (34, 35), and anti-cancer activities (35–37). Its anti-oxidative activity is higher than curcumin itself (38). Our previous studies also discovered that THC has potent anti-angiogenic effects in tumor (39, 40), surpassing those of curcumin (40). However, the effect of THC on angiogenesis in adipose tissue in VHFD-induced obesity mouse model has not been explored. Because THC is known for its anti-angiogenic activity and its ability to suppress tumor growth, we hypothesized that THC could potentially prevent adipose tissue growth by inhibiting angiogenesis. To investigate this, the current study examines the effects of THC supplementation on adipose tissue angiogenesis and the expression of angiogenic factors in VHFD-induced obese mice.

## 2. Materials and methods

### 2.1. Preparation of THC

Ten percent palladium on charcoal was added into a solution of CUR in EtOH. After degassing, the mixture was hydrogenated at ambient temperature for 3 h. The mixture was filtered through a column of cellulose, and the solvent was subsequently evaporated. The crude product was separated via column chromatography using gradient elution of CH_2_Cl_2_-MeOH, resulting in a 70% yield of THC. THC appeared as colorless needles (from CH_2_Cl_2_-n-hexane) with a melting point of 93–94°C. The spectroscopic data (IR, ^1^H-NMR, and mass spectra) of the synthesized THC were consistent with the reported values (29). THC solution in this study was diluted in 1% DMSO in corn oil.

### 2.2. Animals and experimental model

All experimental protocols were reviewed and approved by the Animal Ethics Committee of Thammasat University (approval code 020/2021). Male ICR mice (20–25 g) were purchased for the Siam Nomura International Co., Ltd. and were transported to the Animal Laboratory Center of Thammasat University. The animals were acclimatized under standard laboratory conditions, and allowed access to food and water *ad libitum* for 1 week. After 1 week of acclimation, the mice were divided into three groups: (1) low-fat diet group (LFD group) (*n* = 8); mice were fed of LFD (7% kcal from fat, CP082G, National laboratory Animal Center, Thailand); (2) Very high-fat diet (VHFD) group (*n* = 8); mice were fed of VHFD (60% kcal from fat, MP Biomedicals, United States) and treated with 1% DMSO in corn oil for 6 weeks (VHFD group); (3) VHFD+THC treated group (*n* = 8); VHFD-fed mice were treated with 300 mg/kg of THC (VHFD+THC group). All treatments were given orally every day for 6 weeks. The body weight and energy consumption were recorded weekly. At the end of the treatment, mice were overnight fast and were anesthetized prior to blood collection via cardiac puncture. The serum was prepared for laboratory analyses including fasting blood glucose and lipid profiles. Their visceral white adipose tissues (vWATs) from retroperitoneal, and mesenteric regions were collected and weighed. The total vWATs weight of each mouse was recorded. The relative adipose tissue weight is expressed as the total vWATs per final body weight of each mouse. vWATs were fixed in 10% formalin for further analyses.

### 2.3. Histological analysis

Paraffin embedded vWATs were stained with hematoxylin and eosin (H&E). To determine the average adipocyte size, 100 adipocytes/mouse were measured in 10 random ×10 microscopic fields from 8 mice per group, using the Image J 1.38 software (National Institutes of Health, United States). The number of crown-like structures (CLSs) was measured using a protocol as previously described (41).

### 2.4. Immunohistochemistry for CD31 expression and microvascular density determination

To quantify angiogenesis, microvascular density (MVD) was assessed by immunostaining with the anti-CD31 antibody. The vWAT samples were incubated with primary monoclonal antibody CD31 (Ready to use, DAKO cytomation, United States) followed the protocol described previously (39). The sections were observed under the low power (×40), then the densest area of microvessel sections was selected and captured 3–5 independent fields of view per mouse. The percentage of the CD31 immunoreactivated area to the total area was analyzed by ImageJ 1.38 software (National Institutes of Health, United States).

### 2.5. Immunohistochemistry for angiogenic biomarkers and MMPs

Paraffin sections of vWATs were incubated with primary monoclonal antibody TNF-α (1:100; ab1793, Abcam, United States), primary monoclonal antibody VEGF (Ready to use, DAKO Cytomation, United States), VEGFR-2 (1:200; ab115805, Abcam, United States), MMP-2 (1:500; ab86607, Abcam, United States), MMP-9 (1:500; ab288402, Abcam, United States) at 4°C for 1 h. After rinsed with PBS, the samples were developed by the Envision system/HRP (DAKO Cytomation, United States) for 30 min and substrate-chromogen (DAB) for 10 min at room temperature. The percentage of the TNF-α, VEGF, VEGFR-2, MMP-2, and MMP-9 immunoreactivated area to the total area was calculated using ImageJ 1.38 software (National Institutes of Health, United States).

### 2.6. Serum analysis

Blood was collected through cardiac puncture during euthanization using 2% isoflurane. To obtain serum, the collected blood was allowed to coagulate for 1 h at room temperature and then centrifuged at 3,000 g for 5 min at 4°C. The concentrations of serum glucose, triglyceride (TG), total-cholesterol, low-density lipoprotein cholesterol (LDL-C), high-density lipoprotein cholesterol (HDL-C) in the serum were evaluated by FURUNO Clinical Chemistry Automated Analyzer, URUNO ELECTRIC Co., Ltd., Nishinomiya, Hyogo, Japan.

### 2.7. Statistical analysis

Statistical analysis was performed using IBM SPSS software version 25 (IBM Corp., Armonk, NY, United States). Analysis of variance (ANOVA) in conjunction with Tukey’s *post hoc* test was used to compare among multiple groups, and a difference of *p* < 0.05 was considered to be statistically significant. The data are presented as the means ± standard error of the mean (SEM).

## 3. Results

### 3.1. Effects of THC on parameters related to the VHFD-induced obesity mouse model

**Figure 1:**
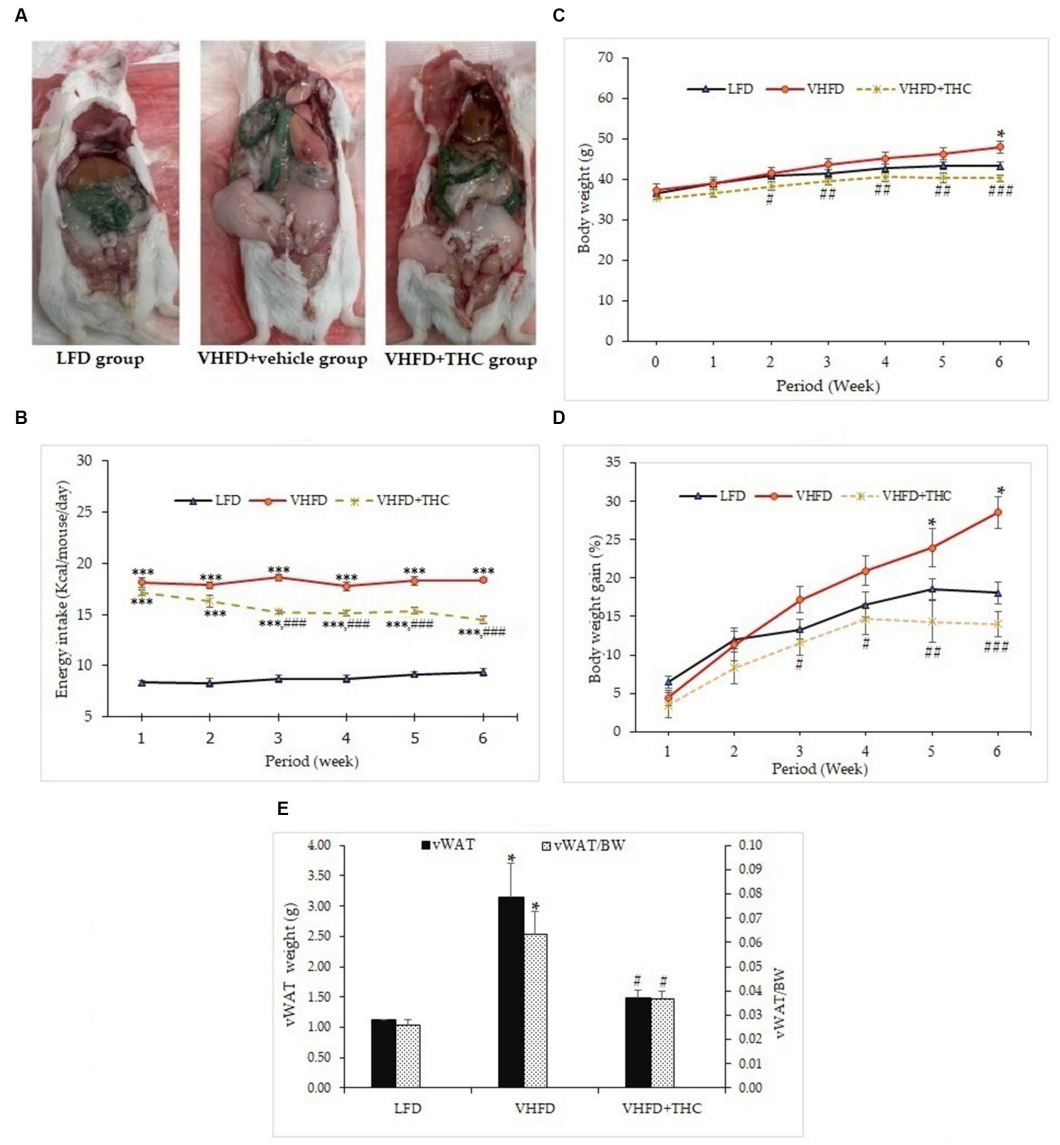
Effects of THC on characteristics of VHFD-induced obese mice: **(A)** autopsy examination of adipose tissue distribution after 6 weeks of treatment; **(B)** energy intake-time curve; **(C)** body weight-time curve; **(D)** body weight gain-time curve; and **(E)** visceral fat weight and relative adipose tissue. Data are presented as mean ± SEM. ^*^p < 0.05 and ^***^p < 0.001 vs. LFD group; ^#^*p* < 0.05, ^##^*p* < 0.005, and ^###^*p* < 0.001 vs. VHFD group.

At the end of the treatment period, autopsy examination revealed that the visceral fat was dramatically increased in VHFD-fed mice compared with LFD-fed mice. The VHFD+THC treated mice showed that the depots of visceral fat were decreased compared with VHFD-fed mice (Figure 1A).

The energy intake of the VHFD groups was significantly higher than the LFD group (*p* < 0.001). Surprisingly, the energy intake was significantly decreased in VHFD-fed mice after 2 weeks of treatment with THC (*p* < 0.001) (Figure 1B).

The body weight of the mice is presented in Figures 1C,D. The untreated VHFD-fed mice displayed significantly higher percent body weight gain and body weight than mice fed with the LFD after 5 and 6 weeks of feeding, respectively (*p* < 0.05). Interestingly, compared to the VHFD group, VHFD+THC treated mice began showing a significant decrease in body weight starting at 2 weeks post-intervention (Figure 1C) while percent body weight gains significantly decreased after 3 weeks of the treatment with THC and these effects continued until the end of the experimental period (Figure 1D).

The visceral fat weight (VF) significantly increased in the VHFD group compared to the LFD group (*p <* 0.05). THC supplementation for 6 weeks significantly reduced VF weight (*p <* 0.05) (Figure 1E). Moreover, the ratio of the visceral fat to the whole-body mass (relative visceral fat weight/body weight, VF/BW) was significantly increased in VHFD group compared to the LFD group (*p* < 0.05) (Figure 1E). However, relative adipose tissue was significantly decreased in the VHFD+THC group than those in the VHFD group (*p* < 0.05), suggesting that THC supplementation prevents the accumulation of the visceral adipose tissue.

### 3.2. Effect of THC on histological changes of the adipose tissue

**Figure 2:**
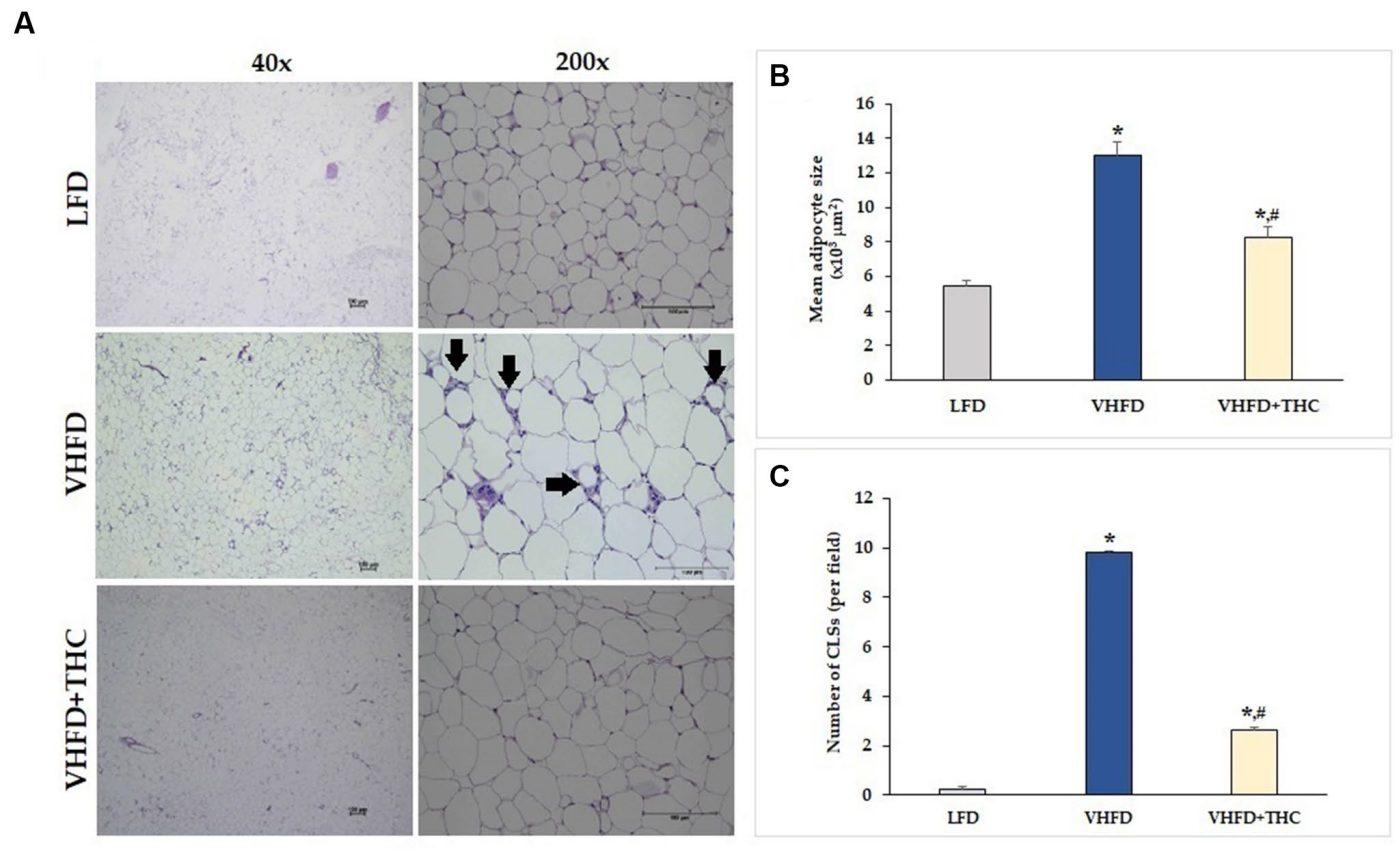
Effects of THC on histological changes of the adipose tissue: **(A)** H&E staining of vWATs; arrows indicate representative structures of CLSs; left column, magnification 40x; scale bar = 100 μm; right column, magnification 200x; scale bar = 100 μm; **(B)** mean adipocyte size; **(C)** number of CLSs in WATs. Data are presented as mean ± SEM. *p < 0.001 vs. LFD group; ^#^*p* < 0.001 vs. VHFD group.

Figure 2A shows H&E staining of adipose tissues. The vWATs of the LFD mice exhibited a normal adipocyte structure without any inflammatory cell infiltration. LFD group also displayed normal adipocyte size, whereas the adipose tissue obtained from the VHFD group showed a significant increase in adipocyte size (*p* < 0.001; Figure 2B) and a large number of CLSs (*p* < 0.001; Figure 2C). THC supplement significantly lowered the adipocyte size and CLSs compared to the VHFD group (*p* < 0.001). However, the adipocyte size and CLSs of THC-treated group mice was still higher than that of LFD group (*p* < 0.001).

### 3.3. Effect of THC on serum level of glucose, total cholesterol, HDL, LDL, and TG

As shown in Table 1, the levels of serum TG and total cholesterol were significantly higher in VHFD-fed than LFD-fed mice. In addition, the levels of LDL and fasting blood sugar in VHFD group were higher than that in LFD group, albeit no significant difference between groups. Interestingly, THC supplementation significantly reduced blood glucose, total cholesterol, and TG. THC-supplemented mice tended to reduce serum LDL than untreated VHFD mice.

**Table 1:** Fasting blood sugar and lipid profiles.

	LFD-vehicle	VHFD-vehicle	VHFD + THC
FBS (mg/dL)	85.37 ± 6.31	102.00 ± 6.49	71.63 ± 8.53^#^
Cholesterol (mg/dL)	87.13 ± 4.86	104.50 ± 2.23*	84.75 ± 5.48^#^
HDL (mg/dL)	72.25 ± 3.89	69.00 ± 4.74	70.50 ± 5.15
LDL (mg/dL)	8.13 ± 1.01	8.25 ± 0.62	6.25 ± 0.37
TG (mg/dL)	107.36 ± 4.15	143.25 ± 4.35***	96.75 ± 7.22^###^

Data are presented as mean ± SEM. *p < 0.05 and ***p < 0.001 for VHFD group vs. LFD group; ^#^*p* < 0.05 and ^###^*p* < 0.001 for VHFD + THC group vs. VHFD group, respectively.

### 3.4. Effect of THC on TNF-α expression

**Figure 3:**
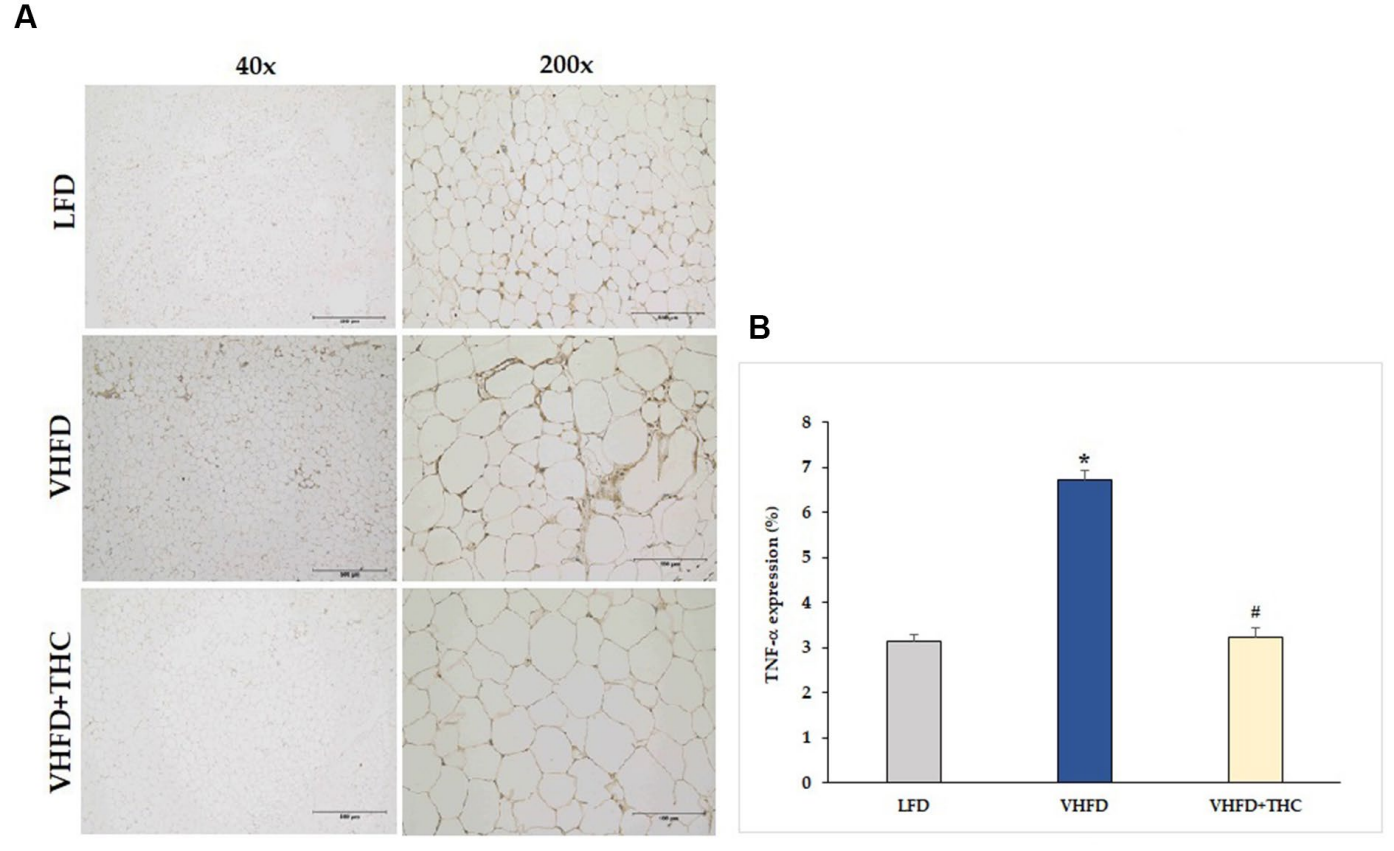
Effects of THC on TNF-α expression: **(A)** representative TNF-α expression; Left column, magnification 40x; scale bar = 500 μm; Right column, magnification 200x; scale bar = 100 μm; **(B)** % TNF-α expression. Data are presented as mean ± SEM. *p < 0.001 vs. LFD group; ^#^*p* < 0.001 vs. VHFD group.

As shown in Figure 3A, TNF-α was over-expressed in VHFD group, whereas its expression was markedly reduced by THC treatment. Figure 3B shows the percentage of positive staining of TNF-α expression. In the VHFD group, TNF-α expression (6.72 ± 0.21%) was significantly higher than in the LFD group (3.14 ± 0.17%) (*p* < 0.001). Interestingly, in the VHFD+THC group, the percentages of positive staining of TNF-α (3.22 ± 0.22%) was significantly lower than those of the VHFD group (*p* < 0.001).

### 3.5. Effect of THC on adipose angiogenesis

**Figure 4:**
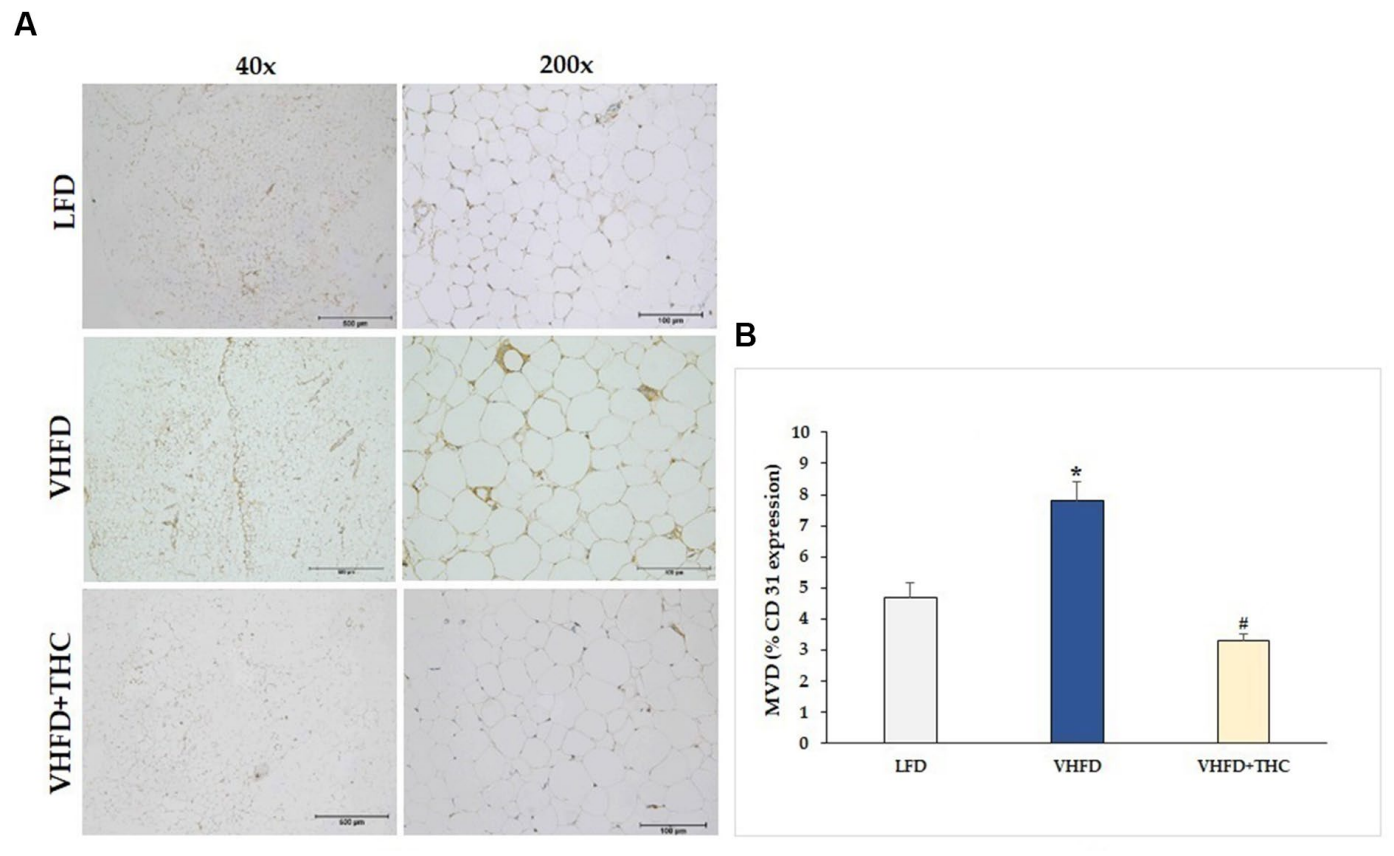
Effect of THC on adipose angiogenesis: **(A)** representative CD-31 expression; Left column, magnification 40x; scale bar = 500 μm; Right column, magnification 200x; scale bar = 100 μm; **(B)** MVD (% CD31 expression). Data are presented as mean ± SEM. *p < 0.001 vs. LFD group; ^#^*p* < 0.001 vs. VHFD group.

The result showed that the adipose tissue of VHFD-fed mice was highly vascularized, whereas THC-treated mice had lower vascularization (Figure 4A). Furthermore, the microvascular density (MVD), as measured by the percent of CD31 expression, was significantly increased in VHFD-fed group (7.82 ± 0.58) compared to LFD group (4.68 ± 0.48) (*p* < 0.001). However, a significant reduction of MVD was detected in the adipose tissue of the THC-treated mice (3.29 ± 0.22) as compared with the nontreated VHFD-fed mice (*p* < 0.001) (Figure 4B).

### 3.6. Effect of THC on angiogenic biomarkers

**Figure 5:**
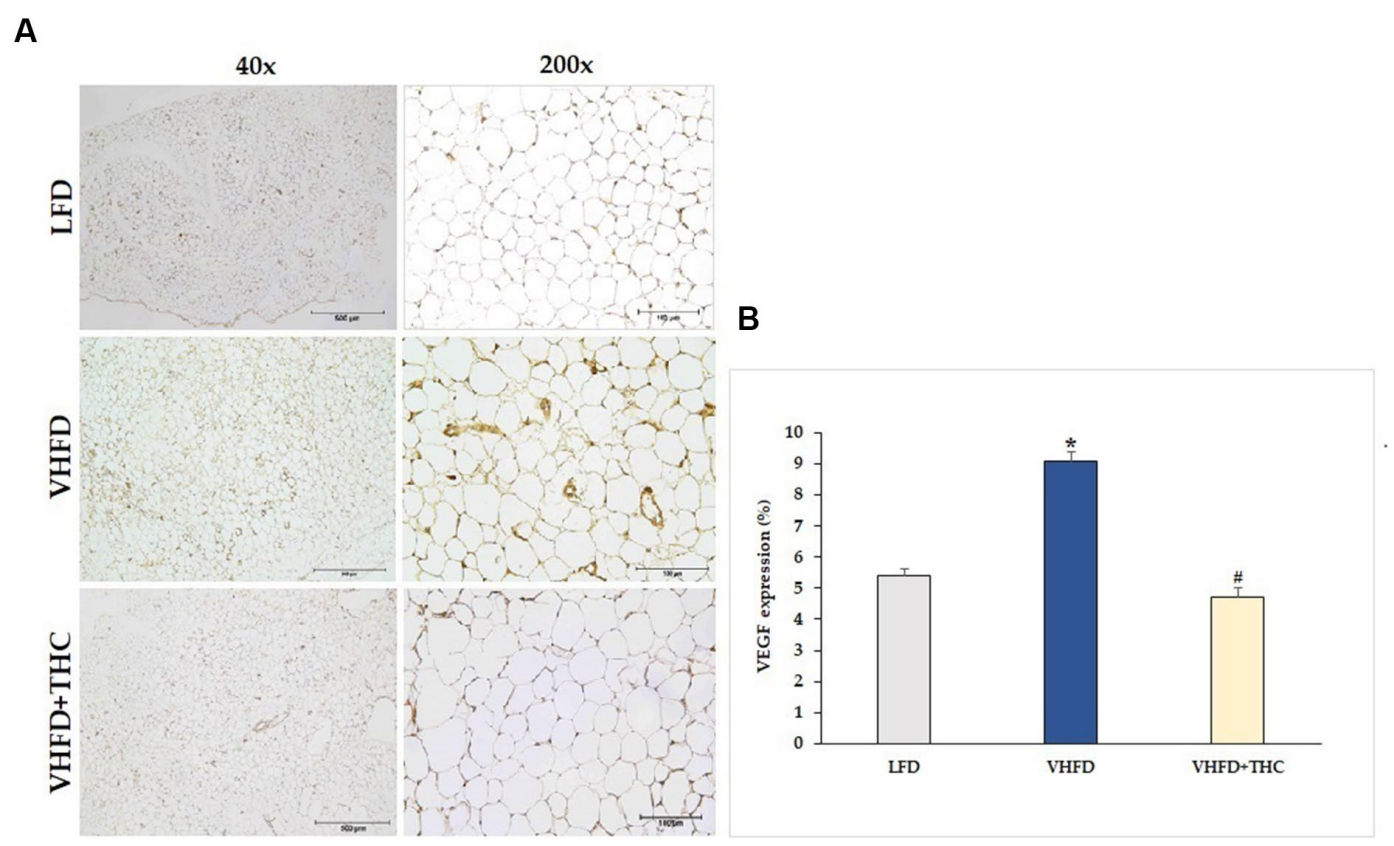
Effect of THC on VEGF expression: **(A)** representative VEGF expression; left column, magnification 40x; scale bar = 500 μm; right column, magnification 200x; scale bar = 100 μm; **(B)** %VEGF expression. Data are presented as mean ± SEM. *p < 0.001 vs. LFD group; ^#^*p* < 0.001 vs. VHFD group.

The use of immunohistochemistry method revealed that the VEGF protein was strongly expressed more in VHFD group than in LFD group. Interestingly, our study demonstrated that THC treated group attenuated VEGF expression (Figure 5A). As shown in Figure 5B, VEGF expression (9.07 ± 0.30%) was significantly higher in the VHFD group than in the LFD group (5.38 ± 0.25%) (*p* < 0.001). However, significant reductions in % VEGF positive staining were found in THC-treated groups (4.71 ± 0.33) (*p* < 0.001).

**Figure 6:**
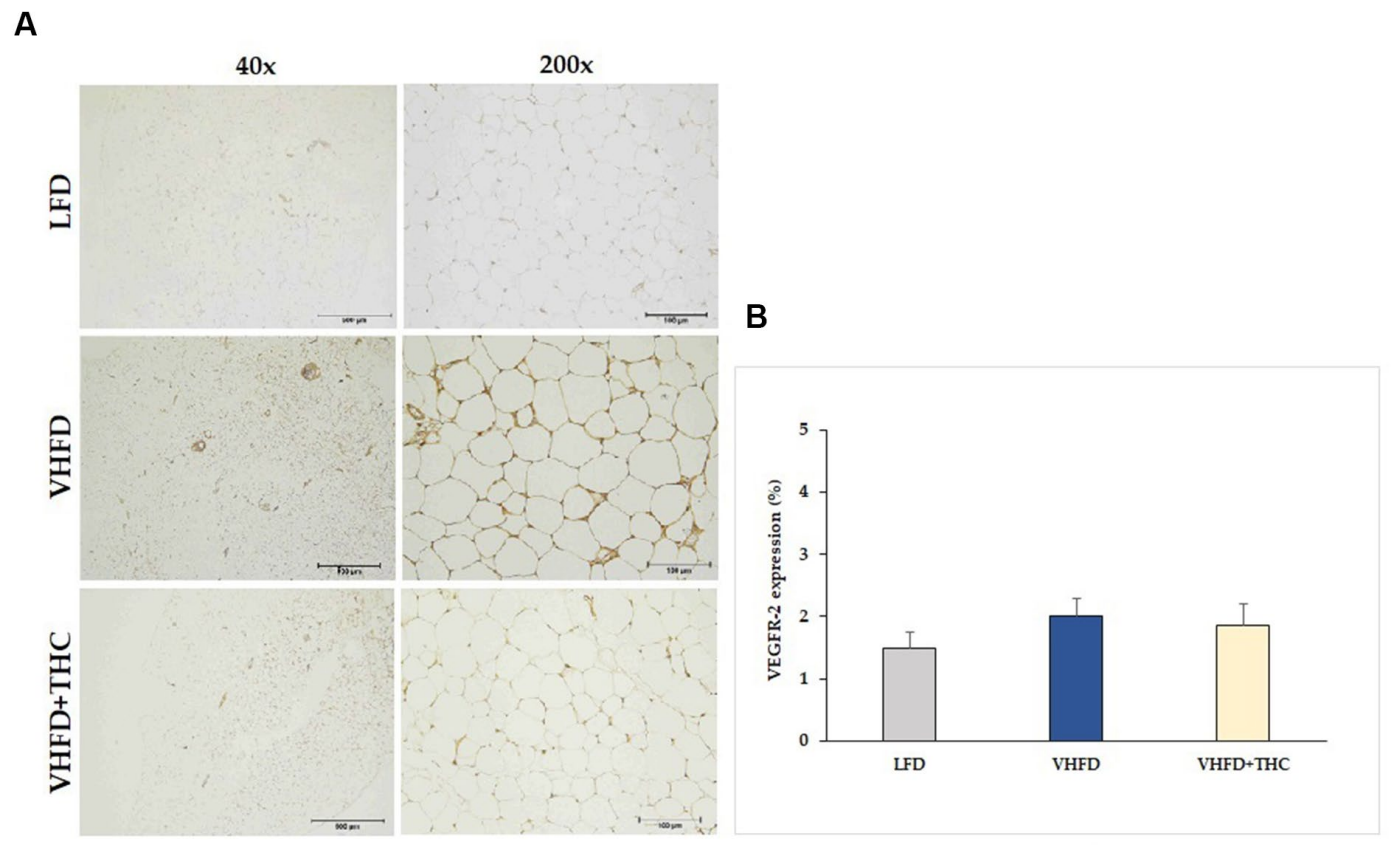
Effect of THC on VEGFR-2 expression: **(A)** representative VEGFR-2 expression; left column, magnification 40x; scale bar = 500 μm; right column, magnification 200x; scale bar = 100 μm; **(B)** %VEGFR-2 expression. Data are presented as mean ± SEM.

The VHFD group also showed stronger VEGFR-2 expression than the LFD group, and the THC treated group showed weaker VEGFR-2 expression than VHFD group (Figure 6A). However, % positive staining intensity for VEGFR-2 expression was not significantly different among groups (Figure 6B).

### 3.7. Effect of THC on adipose MMPs expression

**Figure 7:**
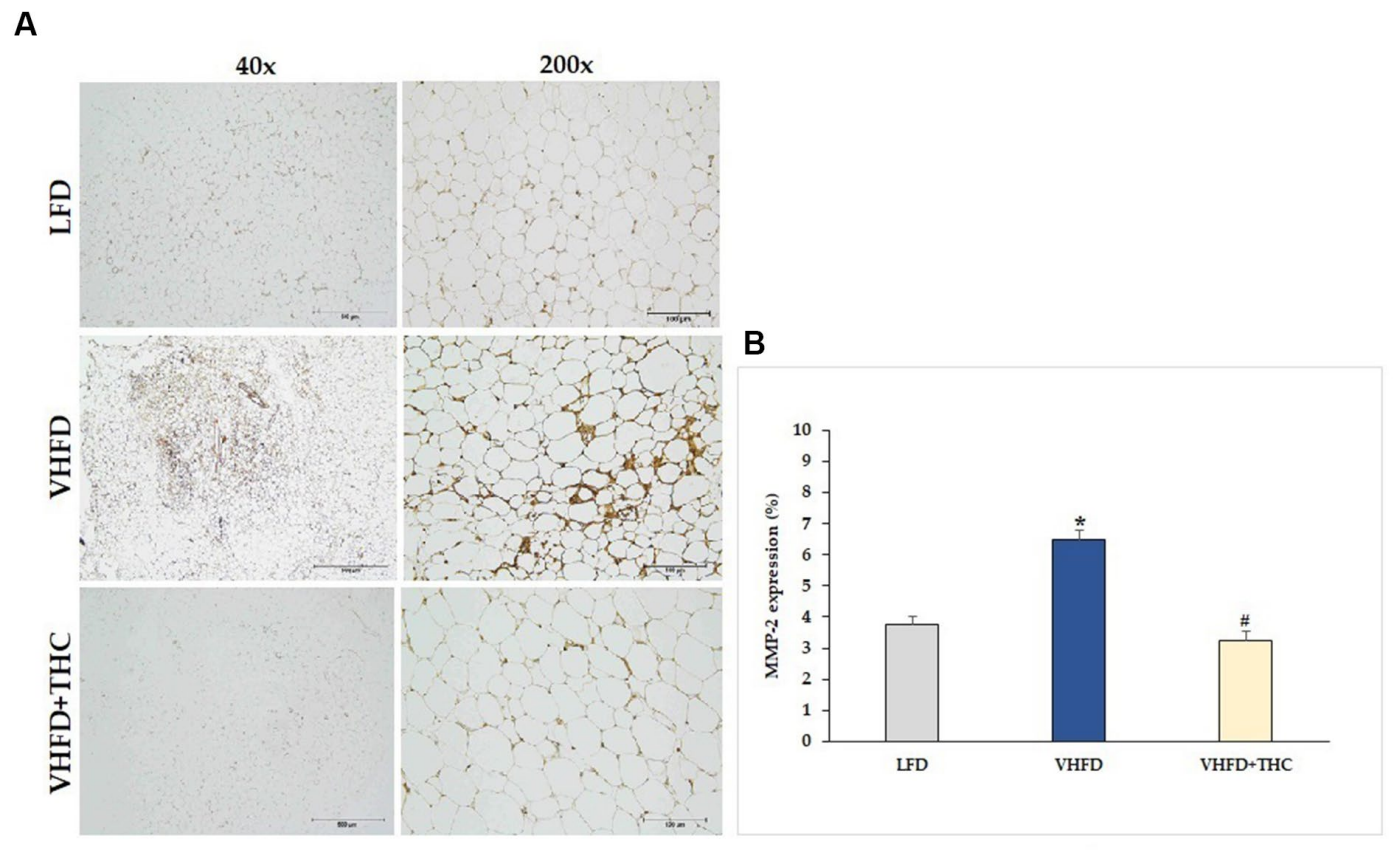
Effect of THC on MMP-2 expression: **(A)** representative MMP-2 expression; left column, magnification 40×; scale bar = 500 μm; right column, magnification 200×; scale bar = 100 μm; **(B)** % MMP-2 expression. Data are presented as mean ± SEM. *p < 0.001 vs. LFD group; ^#^*p* < 0.001 vs. VHFD group.

**Figure 8:**
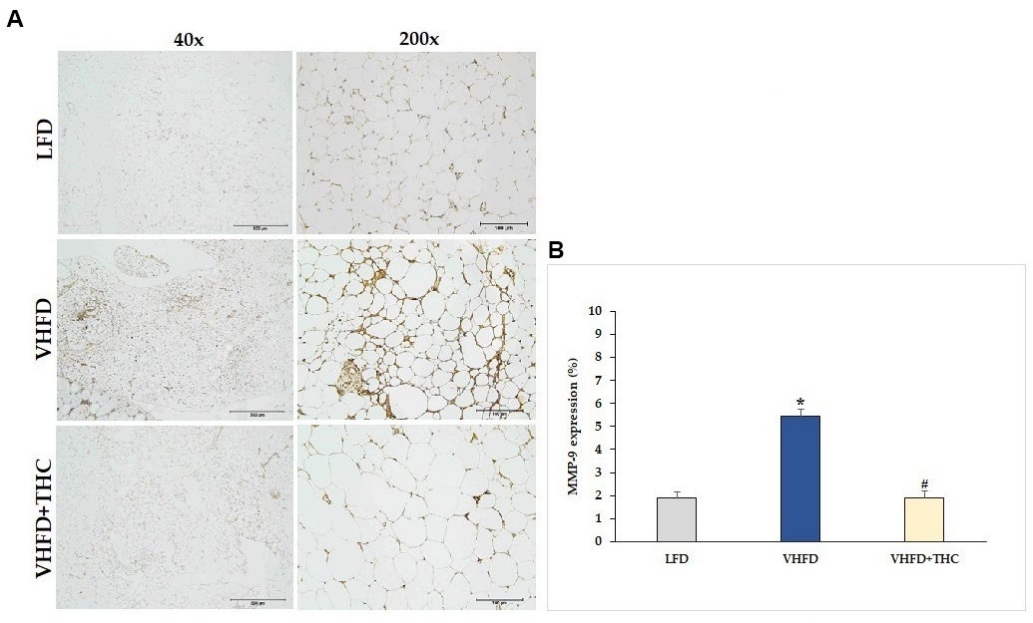
Effect of THC on MMP-9 expression: **(A)** representative MMP-9 expression; left column, magnification 40×; scale bar = 500 μm; right column, magnification 200×; scale bar = 100 μm; **(B)** % MMP-9 expression. Data are presented as mean ± SEM. *p < 0.001 vs. LFD group; ^#^*p* < 0.001 vs. VHFD group.

MMP-2 and MMP-9 expression dramatically increased in VHFD group, whereas their expression was reduced notably after THC treatment (Figures 7A, 8A, respectively). Figures 7B, 8B show the percentage of positive staining of MMP-2 and MMP-9, respectively. In the VHFD group, MMP-2 (6.49 ± 0.34%) and MMP-9 (5.44 ± 0.86%) expression were significantly higher than in the LFD group (MMP-2: 3.77 ± 0.18%; MMP-9: 1.92 ± 1.11%) (*p* < 0.001). Interestingly, in the VHFD+THC group, the percentages of positive staining of MMP-2 (3.24 ± 0.24%) and MMP-9 (1.89 ± 0.64%) were significantly lower than those of the VHFD group (*p* < 0.001).

## 4. Discussion

Due to the faster induction of obesity and stronger metabolic responses, very high fat diet (VHFD) which provides 58–60% kcal fat has recently become a popular alternative to more traditional HFD which provides 40–45% total kcal fat (42). In this study, we used VHFD (60% fat) to induce obesity in the mouse model. We demonstrated significant increases in body weight, body weight gain, visceral fat weight, and relative adipose tissue in the VHFD-fed mice compared with the normal-diet-fed mice (Figure 1). VHFD-fed mice had the elevation of triglycerides, blood sugar, total cholesterol, and LDL cholesterol (Table 1). Moreover, VHFD was shown to promote the accumulation of lipids in mature adipocytes, contributing to hypertrophic adipose tissue expansion. In accordance with our previous study, VHFD intake increased body weight, fat accumulation, and adipocyte size in the intra-abdominal compartment, as well as dysfunctional hypertrophic adipocytes. These adipocytes secrete chemokines that attract immune cells, particularly macrophages, leading to the formation of crown-like structures (CLSs) around inflamed adipocytes. These structures further promote local and systemic inflammatory responses by activating various inflammatory genes, including NF-κB p65, MCP-1, TNF-α, and iNOS (41). The various cytokines, chemokines, and growth factors produced by inflammatory process and inflamed adipocytes also promote the differentiation of preadipocytes, angiogenesis, and inflammation within dysfunctional adipocytes, ultimately resulting in insulin resistance (43). The VHFD-induced obesity mouse model in the present study demonstrated an increased CLSs formation in visceral adipose tissue (Figure 2). Our findings that TNF-α was over-expressed in VHFD-fed mice (Figure 3), indicating the occurrence of the inflammation processes in adipose tissues.

When VHFD-induced obese mice were treated with THC, a significant decrease of body weight and percent body weight gain as well as a reduction of the visceral fat weight and relative adipose tissue were observed. These findings indicate the beneficial effects of THC which can inhibit adipose tissue growth, reduce adipose tissue mass, and prevent body weight gain in obese mice. Interestingly, there was a significant reduction of food consumption in the VHFD+THC mice compared to the untreated VHFD mice, suggesting that the reduction of body mass gain, visceral fat weight and relative adipose tissue in THC-treated mice might be due to lesser energy intake (Figure 1B).

Oral supplementation with THC in VHFD-fed mice could decrease the levels of serum TG, total cholesterol, fasting blood glucose and tend to decrease LDL cholesterol, suggesting the effect of THC on lipid and glucose metabolism. A previous study also reported that THC significantly reduced body weight as well as delayed adiposity, steatosis, hyperglycemia, and insulin resistance in obese mice (44). They showed that THC markedly alleviated steatosis via the downregulation of lipogenesis, the activation of AMP activated protein kinase (AMPK), and the increase of fatty acid oxidation (44). Moreover, elevated blood glucose and insulin resistance were improved by THC possibly via a regulation of the hepatic insulin signaling cascade, gene transcription involved in glucose metabolism, and reduction of macrophage infiltration in the liver and adipose tissue (44). In the present study, THC could significantly reduce inflammatory cell infiltration, as seen by the reduction of CLSs in vWATs (Figure 2) and by the reduction of TNF-α expression (Figure 3) in VHFD-fed mice. These finding emphasizes the potential of THC to mitigate inflammation in adipose tissue, leading to the improvement of metabolic dysfunction, including dyslipidemia and hyperglycemia. Adipocyte hypertrophy in vWATs is known to be closely associated with various metabolic syndromes, including insulin resistance (45). THC might alleviate insulin resistance by inhibiting adipocyte hypertrophy and inflammation in obese mice.

In rapidly expanding adipose tissue, hypoxia emerges as another significant factor influencing vascular growth and remodeling (46). In response to hypoxia, adipose tissues produce hypoxia-inducible factor-1α induced angiogenic factors such as VEGF, leptin, and TNF-α, all of which play roles in regulating angiogenesis (47). Thus, it becomes reasonable to speculate that expansion of adipose tissue is associated with local hypoxia, ultimately contributing to angiogenesis through the induction of multiple growth factors. Moreover, activated macrophages produce potent inflammatory cytokines including TNF-α which could stimulate VEGF secretion (48), supporting that the inflammation of adipose tissues contributes to increased angiogenesis. The progression of obesity has demonstrated clear associations with both angiogenesis and the remodeling of extracellular matrix (ECM) (49). By interfering with the angiogenesis process, it is possible to mitigate the hypertrophy and hyperplasia of adipose tissue. In the present study, the microvascular density in adipose tissue was assessed using an anti-CD31 antibody. As expected, the adipose tissue of mice fed with VHFD was found to be highly vascularized (Figure 4), and a positive correlation was observed between the increased vascular density of visceral adipose tissue and the higher expression of VEGF (Figure 5).

VEGF, an important factor in angiogenesis, is expressed at the highest level in visceral adipose tissue (50, 51). Endothelial cells along with infiltrated inflammatory cells and stromal cells of adipose tissues contribute to VEGF production. The VEGF families currently includes VEGF-A, -B, -C, -D, −E, -F, and PlGF (placental growth factor), which bind in a distinct pattern to three structurally related receptors namely VEGFR-1, −2, and −3 (14). The VEGF-A/VEGFR-2 pathway plays a central role in angiogenesis. Ejaz et al. demonstrated that supplementing the high-fat-diet of mice with curcumin could lower body weight gain, the growth of adipose tissue, and angiogenesis in the adipose tissue thru the reduced expression of VEGF and VEGFR-2 (23).

In the present study, supplementation of the VHFD with THC 300 mg/kg for 6 weeks markedly reduced microvascular density within adipose tissue, accompanied by a reduction in the expression of VEGF (Figure 5). Notably, at the dose of 300 mg/kg daily, THC has exhibited a more potent anti-angiogenetic activity in mice with hepatocellular carcinoma compared to CUR (40). The results are remarkable considering the relatively short duration of 6 weeks in comparison to the earlier study by Ejaz et al. (23). In their work, Ejaz et al. demonstrated that CUR inhibited various processes including angiogenesis, adipogenesis, differentiation, apoptosis, and the expression of genes involved in lipid and energy metabolism in 3 T3-L1 adipocytes. Furthermore, they observed effects on body weight gain and adiposity in mice fed a high-fat diet (22%), with CUR supplementation at the dose of 500 mg/kg diet for 12 weeks. In the current study, THC has shown efficacy at a lower dose of 300 mg/kg. Notably, this dose is even lower than the effective dose of CUR. These findings highlight that THC is more efficient than its parent compound, CUR, in suppressing adipose angiogenesis.

The chemical structure of THC closely resembles that of CUR (Supplementary Figure S1); however, it lacks the double bonds in the central seven-carbon chain within the molecule. The characteristic color of turmeric stems from the presence of two conjugated double bonds in CUR, whereas THC appears as an off-white color due to the absence of the α, β-unsaturated carbonyl moiety. THC is soluble in organic solvents such as ethanol, dimethyl sulfoxide (DMSO) and dimethylformamide (DMF). Specifically, its solubility in these solvents is approximately 0.25, 5, and 10 mg/mL, respectively. THC is stable in aqueous solution at a pH of 8.0, with no detectable decomposition within 2 h. However, in an acid environment, it might be slightly decomposed (38). Moreover, THC exhibits a slower degradation rate than CUR, with terminal half-life of 813 min in cell culture medium and 232 min in plasma (52). An *in vitro* study revealed the conversion of CUR into THC, with a yield of 90% per day (53).

The superior oral absorption of THC has also been demonstrated in *in vivo* studies. Following a 4-week regimen of daily oral THC administration, higher levels of free THC and its conjugates (sulfates and glucuronides) were detected in the liver and serum compared to mice given CUR (54). These studies suggested that THC exhibits superior gastrointestinal absorption in comparison to CUR (55). THC demonstrated enhanced solubility, stability and bioavailability compared to CUR (38, 52, 56), resulting in its greater potency. In terms of its biological activities, THC exhibited antioxidant and antidiabetic effects that surpass those of CUR (30, 32–34, 57). The potent antioxidative property of THC is likely related to its beta-diketone moiety (32).

Obesity is characterized by chronic low-grade inflammation accompanied by a persistent increase in oxidative stress (58). THC has demonstrated a greater inhibitory effect on lipid peroxidation in red blood cell membrane, in comparison to CUR. Moreover, this study suggested that the β*-*diketone moiety of THC exhibited antioxidant activity through cleavage of the C–C bond at the active methylene carbon situated between two carbonyls in the β*-*diketone moiety. In fact, THC exhibits antioxidant activity 4–6-fold stronger than trolox, the water-soluble derivative of vitamin E (33). Increasing consumption of THC in the diet should result in beneficial effects in individuals with obesity.

THC has demonstrated low toxicity in several *in vivo* studies (40, 59). Our previous study showed that a 3,000 mg/kg oral dose of THC administered to nude mice for 14 days exhibited no signs of an acute toxicity (40). In the sub-chronic toxicity study, rats fed THC up to 400 mg/kg for 90 days did not show mortality or adverse effects (60). Furthermore, in 13-month-old mice, long-term consumption of 300 mg/kg THC resulted in a longer average lifespan (59). Collectively, these studies underscore the potential safety of THC for both pharmaceutical and nutraceutical applications.

It is noteworthy that in the present study, the percentages of VEGFR-2 expression were also higher in VHFD-fed mice than the controls, and THC treatment tend to decrease VEGFR-2 expression (Figure 6), but these differences were not statistically significant. These findings suggest that the anti-angiogenic effect of short-term THC supplement was due to the downregulation of VEGF expression but not VEGFR-2 expression.

**Figure 9:**
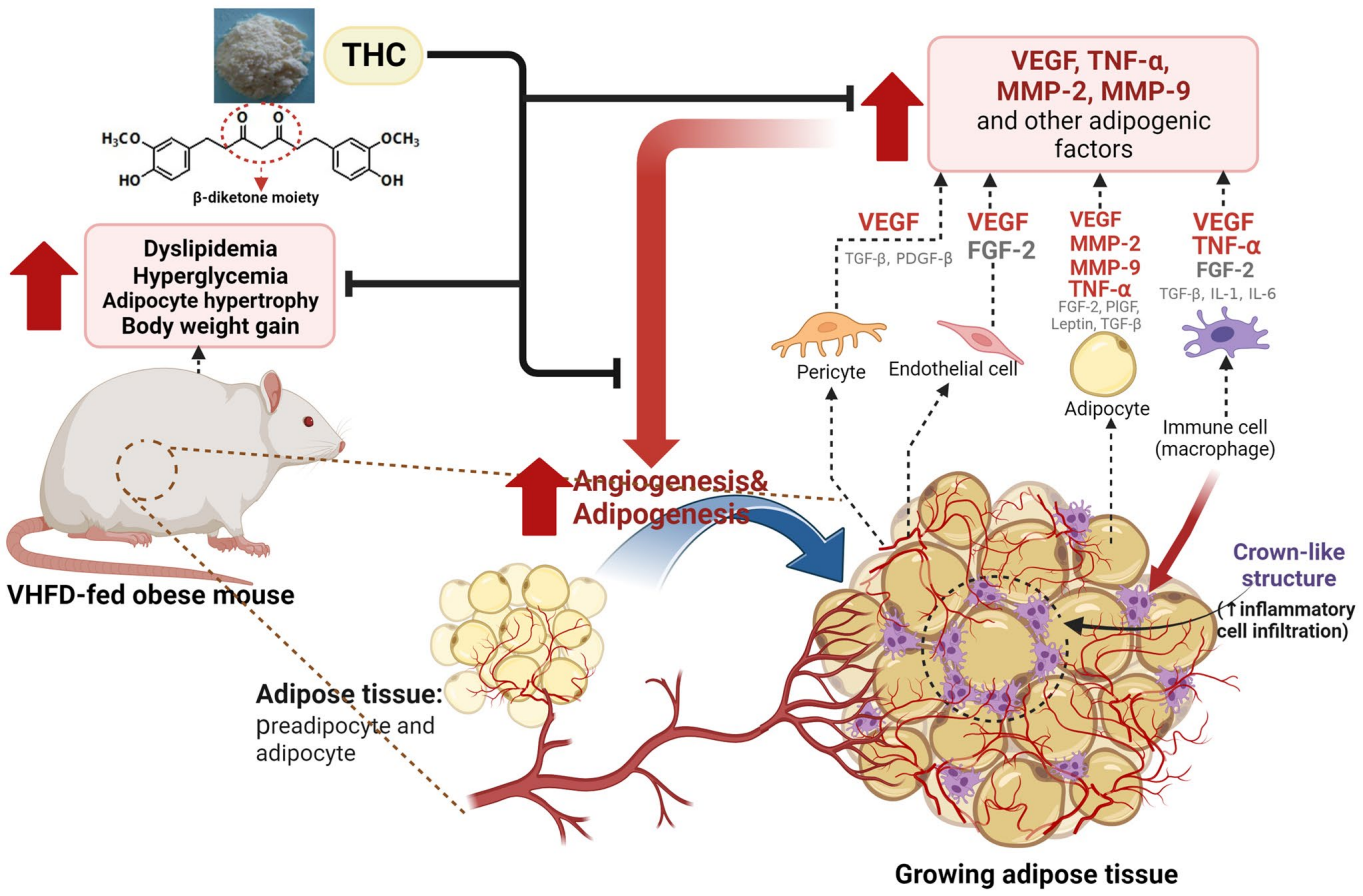
Proposed model for the effect of THC on adipose angiogenesis in very high-fat diet-induced obesity mouse model. THC treatment resulted in decreased weight gain, visceral fat weight, adipose tissue mass, and inflammatory cell infiltration, as indicated by the reduction of crown-like structures (CLSs) in visceral white adipose tissues of VHFD-induced obese mice. This reduction led to an improvement of metabolic dysfunction, including dyslipidemia and hyperglycemia. Additionally, THC exerts anti-obesity activity by inhibiting angiogenesis through the reduction of angiogenic factors (VEGF and TNF-α) and extracellular matrix remodeling through the reduction of MMP-2, and MMP-9.

Besides vascular growth factors, adipocytes release several MMPs which play a key role in angiogenesis. Adipose-tissue MMPs, including the prominent MMP-2 and MMP-9, could potentially affect preadipocyte differentiation and microvascular maturation by modulating ECM (49). This suggests that the regulation of obesity might involve the synergistic interplay between angiogenesis and MMPs. Moreover, MMP-9 can release the matrix bound VEGF, leading to the process of neo-vascularization (61). In this study, an increased expression of both MMP-2 and MMP-9 was found in the adipose tissues of mice fed a VHFD (Figures 7, 8). Interestingly, mice treated with THC exhibited a significant decrease in the expression of MMP-2 and MMP-9, suggesting that THC may inhibit adipose angiogenesis through the downregulation of MMP-2, MMP-9, and angiogenic factors, TNF-α and VEGF expression. Finally, the mechanisms underlying the impact of THC on adipose tissue angiogenesis and obesity are summarized in Figure 9.

While various adipokines, including transforming growth factor-β (TGF-β), IL-1β, IL-6, and IL-8, leptin, insulin, insulin-like growth factor-1 (IGF-1), have been implicated in the inflammatory processes associated with adipose angiogenesis and insulin resistance, our current investigation focused on TNF-α, VEGF, and MMPs on adipose angiogenesis. We believe that future investigations with a boarder spectrum of adipokines and the incorporation of long-term research approach are essential to confirm and strengthen our findings.

## 5. Conclusion

The present study highlights the short-term beneficial effects of THC (the major metabolite of curcumin) that can suppress angiogenesis in adipose tissue via the reduction of angiogenic factors (VEGF and TNF-α) and extracellular matrix remodeling through the reduction of MMP-2, and MMP-9 in the VHFD-induced obese mice model. This anti-angiogenic activity, together with the suppression of food consumption and adipose inflammation, appear to be responsible for the lower body weight gain, visceral fat weight, relative adipose tissue, and, ultimately, the alleviation of dyslipidemia and hyperglycemia in the obese mice. Overall, our study demonstrated the potential benefit of THC in mitigating obesity and associated metabolic disorders as well as elucidating the suppression of angiogenesis in adipose tissue as one of its underlying mechanisms.

## Data availability statement

The original contributions presented in the study are included in the article/Supplementary material, further inquiries can be directed to the corresponding author.

## Ethics statement

The animal study was approved by the Animal Ethics Committee of Thammasat University. The study was conducted in accordance with the local legislation and institutional requirements.

## Author contributions

BY, US, PP, NS, ND, NP, NMa, and NMu designed the study, conceptualized the methodology and validation, and contributed to data analysis. BY, US, PP, and PT wrote original draft, edited, and revised the manuscript. BY and CC contributed to resources. BY, US, PP, NS, and NP contributed to funding acquisition. All authors have read and agreed to the published version of the manuscript.
