# Supplementary figures and images for: Short term effect of tetrahydrocurcumin on adipose angiogenesis in very high-fat diet-induced obesity mouse model
Source: Front Nutr. 2023 Oct 9;10:1221935. doi: 10.3389/fnut.2023.1221935 (PMC10591188; doi:10.3389/fnut.2023.1221935)

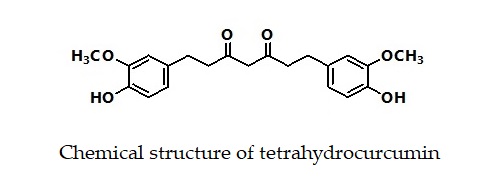

Supplement: Supplementary file 1 [file Image_1.jpg]
